# Access to healthcare services for people with non-communicable diseases during the COVID-19 pandemic in Ibadan, Nigeria: a qualitative study

**DOI:** 10.1186/s12913-023-10278-0

**Published:** 2023-11-09

**Authors:** Lucia Yetunde Ojewale, Ferdinand C. Mukumbang

**Affiliations:** 1 Department of Nursing, University of Ibadan, Ibadan, Oyo State Nigeria; 2 Department of Global Health, University of Washington, Seattle, WA USA

**Keywords:** Non-communicable diseases, COVID-19, Health care, Access, Delay, mHealth

## Abstract

**Background:**

Desirable outcomes for people with non-communicable diseases (NCDs) are achieved when they access routine monitoring and care services. Expectedly, the COVID-19 pandemic severely impacted access to healthcare services, leading to poor health outcomes among people with NCDs. We aimed to [1] explore the delays in accessing healthcare services and [2] understand alternative actions adopted by people with NCDs to overcome these delays.

**Methods:**

We conducted an exploratory qualitative research guided by the “Three Delays” model to unpack the barriers to healthcare access for people living with NCDs in Ibadan, Nigeria. The “Three Delays” model conceptualizes the reasons for negative/adverse healthcare outcomes related to the patient’s decision-making to seek healthcare, reaching an appropriate healthcare facility, and receiving adequate care at the healthcare facility. Twenty-five (25) people with NCDs were purposively selected from the University College Hospital’s medical outpatient department to participate in in-depth interviews. Interview recordings were transcribed verbatim and analyzed using a deductive-inductive hybrid thematic analysis.

**Results:**

At the level of individual decision-making, delays were related to fear of contracting COVID-19 in the hospital (considered a hotspot of the COVID-19 pandemic). Regarding reaching an appropriate healthcare facility, delays were mainly attributed to the intra- and inter-city lockdowns, limiting the movements of persons. For those who successfully arrived at the healthcare facilities, delays were related to the unavailability of healthcare professionals, prioritization of COVID-19 patients, and mandatory adherence to COVID-19 protocols, including COVID-19 testing. To overcome the delays mentioned above, people with NCDs resorted to (i) using private healthcare facilities, which were more costly, (ii) using virtual consultation through mobile phone Apps and (iii) self-management, usually by repeating previously prescribed prescriptions to obtain medication.

**Conclusion:**

Pandemic conditions provide unique challenges to people with chronic illnesses. Recognizing the need for continuous access to monitoring and care services under such conditions remains critical. Alternative health service provision approaches should be considered in pandemic situations, including remote healthcare services such as Mobile health apps (mHealth) that can help manage and prevent NCDs.

**Supplementary Information:**

The online version contains supplementary material available at 10.1186/s12913-023-10278-0.

## Introduction

Non-communicable diseases (NCDs) are the leading cause of death and disability globally, accounting for about 70% of all deaths [1, 2]. Sub-Saharan Africa has been experiencing increased NCD incidence in the last twenty years due to an increased prevalence of NCD risk factors [1]. In Nigeria, NCDs accounted for 29% of all deaths in 2016, and the probability of premature deaths from NCDs is higher than the global trend [3]. In one of the cities in Southwestern Nigeria, hypertension prevalence was as high as 35%, with an even higher percentage (47%) suffering from dyslipidaemia, while 4.6% had diabetes mellitus [4].

A chronic medical condition contributes to COVID-19 morbidity and mortality [5, 6]. The COVID-19 pandemic reportedly contributed to poor access to health care as resources and personnel were diverted to managing the pandemic [7]. Such resource reallocation was often detrimental to providing care for other disease conditions, including NCDs. Before the COVID-19 pandemic, low- and middle-income countries (LMIC) already suffered from inadequate human resources for health, insufficient funding, poorly organized health systems, and incompetent leadership [8]. Consequently, in the era of the COVID-19 pandemic, attaining sustainable development goal 3 to ensure equitable access to quality health care globally became less attainable for many LMICs [9].

NCDs are long-term conditions self-managed by patients but require consistent monitoring by healthcare providers. Routine medical checks and medication adherence are essential requirements for NCD management [10]. At the beginning of the COVID-19 pandemic, Okereke et al. [9] forecasted a disruption in NCD services, including a delay in NCD detection and discontinued or interrupted treatment. This prediction was confirmed by the World Health Organisation’s (WHO) rapid assessment a few months after the pandemic, revealing that only 72% of developed countries and 42% of LMIC considered COVID-19 in their readiness and response plan [11].

The literature has reported several ways the COVID-19 pandemic affected the healthcare access of people with NCDs. Some of these include increased self-medication [12] and disruptions in follow-up services [13], poor access to medications and emergency healthcare services, and postponement of NCD appointments were also reported [14, 15]. Consequently, people living with NCDs reported suffering from a deterioration of their chronic health condition [16, 17].

Many studies have been conducted in Africa to examine the impact of the COVID-19 pandemic on maternal and child health services [18, 19], HIV-hypertension integrated care [20], HIV services including antiretroviral therapy [21–23], and sexual and reproductive health services [24]. A review conducted by Adejumo [25] reported barriers to accessing care, including dialysis services, besides financial difficulties among patients receiving treatment for renal problems. Nwoke et al. [17] reported reduced income and subsequent difficulty buying medications among people with chronic medical conditions. However, to our knowledge, there is limited information based on the accounts of people with NCD accessing follow-up care during the pandemic. To this end, we sought to (1) describe the experience of people living with chronic illness in accessing their usual healthcare services using the “Three Delays” model and (2) identify the alternative healthcare services used by these patients for their healthcare needs.

## Conceptual framework

We underpinned this study on the “Three Delays” model described by Thaddeus et al. [26]. The “Three Delays” model conceptualizes the reasons for negative/adverse healthcare outcomes related to the patient’s decision-making to seek healthcare, reaching an appropriate healthcare facility, and receiving adequate care at the healthcare facility. The “Three Delays” model was designed in response to the causes of poor outcomes in maternity care. It has, however, been applied to unpack the causes of adverse health outcomes of other disease conditions, including the COVID-19 pandemic [27]. The “Three Delays” in the model include (1) delays in deciding to seek care by the patient/family members, (2) delays in accessing healthcare facilities which are influenced by accessibility factors such as travel time from home to facility, distribution of facilities, availability, and cost of transportation, among others, and (3) delays in receiving adequate care which may be influenced by shortages of supplies, equipment, and trained personnel, adequacy of the referral system and competency of available personnel [26].

## Methods

### Study design

We employed an exploratory qualitative design. Exploratory research designs allow researchers to summarise events that people or groups have encountered in simple terms [28]. The goal was to obtain relevant data to help us unpack people’s experiences with chronic medical conditions during the COVID-19 pandemic using the “Three Delays”.

### Study setting

This qualitative study is part of a larger study titled “Exploration of COVID-19 health-related issues among people living with chronic health conditions in Ibadan”. A previous qualitative study investigated the factors contributing to COVID-19 vaccine hesitancy among individuals who indicated they were not willing to receive the COVID-19 vaccine [29]. The larger study took place in Ibadan, the capital of Oyo State, one of the 36 states of Nigeria.

Ibadan is the second-largest city in Nigeria, with a population of about 3,649,000 as of 2021 [30]. Ibadan has several primary healthcare centres and general hospitals located strategically, with two teaching hospitals, the University College Hospital (UCH) and Adeoyo Maternity Teaching Hospital (AMTH). AMTH caters mainly for maternal/child health issues, while UCH has a much larger healthcare service provision scope. The UCH is an 850-bed capacity hospital that provides various services, including clinical (both in-patient and outpatient) healthcare services, research, and training of healthcare personnel at undergraduate and postgraduate levels.

### Sampling technique and study sample

Participants were recruited to participate in the study through a purposive sampling strategy. Participants were met in person at the UCH medical outpatient department. The clinic operates daily during the week, from Mondays to Fridays, with different medical issues being treated on other clinic days. While the patients waited to obtain their NCD-related services, we introduced the study conveniently to those in the waiting area. We asked them whether they would be open to participating in the study. Participants were considered eligible if they met the following criteria: (1) older than 18 years; (2) had a chronic non-communicable disease condition for more than six months following an official medical diagnosis; (3) willing to consent and participate in the study; and (4) registered in UCH before the pandemic (5) healthy enough physically and mentally to participate in the study. Table 1 displays the characteristics of the study participants. Potential participants were excluded if they were pregnant, very ill, or unconscious while trying to access health care during the COVID-19 pandemic.

**Table 1:** Sociodemographic characteristics of study participants

Variable	Categories	n	%
**Gender**	Male Female	12 13	52 48
**Age – years**	20–40 41–60 61–80 > 80	05 12 67 01	20 48 28 04
**Ethnic group**	Yoruba Others (Igbo, Edo, Ibibio)	22 03	88 12
**Religion**	Christianity Islam	20 05	80 20
**Marital status**	Single Married Divorced	02 22 01	08 88 04
**Educational level**	No formal Primary Secondary Diploma First degree Postgraduate	01 03 04 06 07 04	04 12 16 24 28 16
**Occupation**	Civil service Trading Retiree others	05 09 06 05	20 36 24 20
**Health condition**	Hypertension Diabetes mellitus Hypertension and DM Chronic kidney disease Sickle cell anaemia Others	10 06 03 02 02 02	40 24 12 08 08 08
**Illness duration** **(years)**	1–10 11–20 > 20	14 05 05	60 20 30

Three more participants who matched the eligibility requirements but opted out cited various reasons, including a lack of time or interest in the study. Participant selection and thematic data analysis were conducted in tandem with the participant selection process. Thematic saturation, when all the constructs that comprise the “Three Delays” model construct are fully reflected by the data, determined our selection of 25 participants [31]. The thoroughness of both the data collection and analysis provided rigour to our saturation.

### Study context

In-depth interviews were conducted in April 2021. At the time, most of the structures and activities in the hospital had almost returned to operating as in the pre-COVID era, although the number of patients seen per day was reduced. COVID-19 measures were also observed, including the compulsory use of facemasks, social distancing, and consultation in open areas.

### Data collection

Data were obtained using a semi-structured interviewing guide (Additional file 1), developed based on a review of the related literature. The interview guide was initially tested with five participants, and it was deemed effective in eliciting the required and desired responses after listening through the recordings of the final interview. LYO, a doctorate in nursing science whose research focuses on the care of patients with chronic health issues, conducted face-to-face interviews in a private section of the clinic. Consultative meetings were held with FCM (a skilled researcher in global health and qualitative research methods) after each interview to assess the data collection process and the emergence of themes to assess data saturation.

The nurse in charge of the medical outpatient clinic announced the interviewer’s presence and intentions to the patient in the waiting area before they were approached for potential participation in the study. The introduction provided information about the researcher’s occupation and the purpose of the study. During her one-on-one discussion with the patients, the interviewer restated the study’s objective—to ascertain access to health care.

The interview guide included questions on the study participants’ underlying health condition and a section to record their sociodemographic details. Some questions in the interview guide include: “Can you tell me about your experience and how life has been for you since COVID-19 started in Nigeria? What was access to healthcare like at UCH during the pandemic? (Probe: did you have your usual clinic appointment?) How did you manage your health during the COVID-19 lockdown? Would you say your health was affected because of the lockdown?

The interview guide was used for consistency throughout all interviews and to ensure a systematized investigation of the phenomenon under consideration. Using the participants’ initial responses to the questions in the interview guide as a starting point, the interviewer probed for further details based on the initial responses. The interviews lasted between 15 and 20 min and were audio recorded. Following every discussion, field notes were taken.

### Data management

A bilingual researcher fluent in English and Yoruba with a master’s degree in public health transcribed the recordings verbatim. No names or other participant-identifying details were recorded during the transcription process. The transcribed texts were entered into Dedoose in Word format and prepared for analysis.

### Data analysis

We conducted a thematic content analysis, a strategy for summarizing qualitative data, attaching meaning, and providing interpretation. It involves creating and selecting codes to generate themes—a thread of underlying meanings within which similar data bits can be found [32]. A hybrid of deductive and inductive data analysis approaches was adopted. The “Three Delays” model directed the overall data analysis and formed the basis of the deductive analysis. Allowing the themes and sub-themes to ‘naturally’ emerge from the transcripts through the coding of the transcripts constituted the inductive aspect of the data analysis.

As the first step (reducing the data), codes were independently generated by the two authors. Following the independent coding, they worked on discursively developing the sub-themes and themes. Both authors also iteratively compared and categorized code clusters into subthemes and subsequent themes concerning the “Three Delays” model.

### Rigor and trustworthiness

We used three approaches to determine rigour and trustworthiness. First, we reviewed the literature rough to develop the interview guides. Then, using a pilot interview, we confirmed that the data collection tool could elicit the essential information from the participants and, consequently, the necessary data to answer the research question.

Second, as the study progressed, the researchers convened several meetings to discuss and adjust the data collection and analysis approaches. This process was critical to determining thematic saturation concerning the “Three Delays” model.

Third, the two authors conducted the data analysis. Although both authors worked independently to complete the initial open, axial coding, iterative and discursive sessions allowed them to classify the sub-themes and themes, identify themes, and then fit the resulting constructs into the Delay model. We documented the whole research process to have an audit trail of the decisions made and their reasons.

## Results

The results are presented in two parts to address the two study objectives. These aims are (1) to describe the experience of people living with chronic illness in accessing their usual health care services using the “Three Delays” model, (2) to describe the alternative health care services utilized by people with chronic conditions during the COVID-19 pandemic.

### Experiences in accessing usual healthcare services

**Fig. 1:**
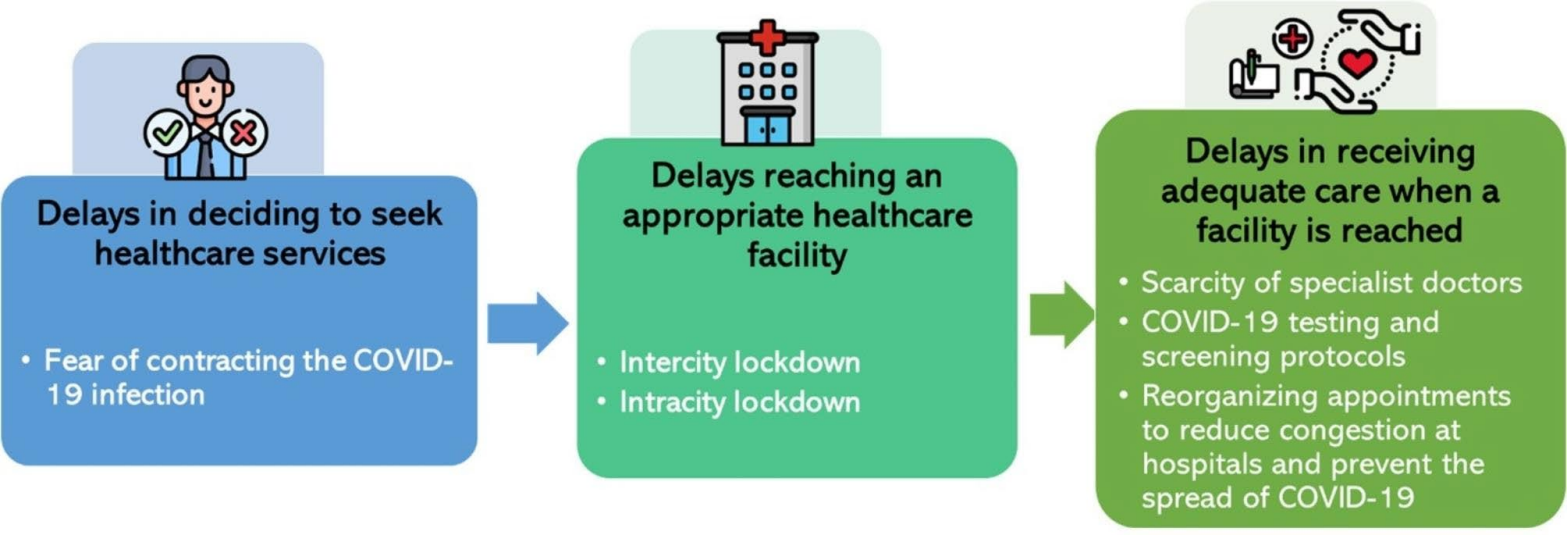
A representation of the themes on the “Three Delays” model

We unpacked the experience of people with chronic illness in accessing their usual healthcare services using the “Three Delays” model. The themes that emanate from our data are represented in Fig. 1.

Regarding seeking care, some patients decided not to visit the hospital due to the fear of contracting the SARS-COV-2 virus. One of the participants explained:

I was not able to come for treatment. For more than a year, [I] have not been here. [I] have not been able to come for a checkup because of COVID-19, am afraid [R15, Female, in her 50s, Lupus erymathosus].

Some patients delayed visiting the hospital due to inter-city or intracity lockdown measures, preventing travels between the state of residence and the city where their usual specialist hospital for receiving treatment was located.

No movement. I live in Lagos [and usually receive treatment in Ibadan]. We have our private family doctor; when I was having malaria, complications, this and that, I went to the doctor in the private hospital where I was admitted for one week [P21, Male, in his 50s, diabetes and chronic kidney disease].

When they said there was a lockdown and everybody should stay home and avoid going out unnecessarily, I did not have a problem. I stayed at home [P25, Female, in her 70s, hypertension].

Patients experienced delays in receiving adequate treatment at the health facility in different ways. Despite distressing symptoms, some were delayed at the emergency service to be tested for COVID-19. One of the participants expressed her experience in the following way:

I was already in pain; I couldn’t walk, so they said I should go and do something when I was already in pain. On a normal day, they would admit immediately. Then, they will start giving you a drip or whatever medication they would administer. They will start giving you, but on that day, they were like, I should go and start doing some other test [COVID-19 test], I was like this, this is not normal [P6, Female, in her 30s, Sickle cell haemoglobinopathy].

Others experienced delays in accessing healthcare after arriving at the hospital. They were told to return later because the hospital was focused on managing COVID-19-related issues, which were considered a priority at the time.

No, there was a particular interval, at a particular time, … they shifted our date of coming for visitation because of the COVID of a thing, yes [P20, Male, in his 60s, hypertension].

Some patients felt it became generally challenging to access healthcare workers compared to pre-COVID-19.

COVID-19 was (a) tug of war for us. We that have one ailment or the other, because it was not like before, that, any slightest complaint, we easily come to the hospital and lodge complaint, but now, before you can see any doctor here, it (is) difficult, so it affected our health [P12, Female in her 60s, diabetes].

Yeah, at the first instance [during COVID-19], it was [difficult to access healthcare workers] because we could not be attended to at the hospital, especially this UCH I am using at that first stage. You find out that when you come here, they tell you that health workers are working, but you will not see them if you come here. That is the truth of the matter [P2, Female, 30s, hypertension].

### Alternative healthcare services

Some participants suggested using alternative healthcare services to overcome some of the delays they experienced. These were classified as (i) use of private health care facilities, (ii) virtual consultation, and (iii) self-medication.

#### Use of private health facility

During the COVID-19 pandemic, some patients with NCDs resorted to accessing care in private care facilities. One of the participants stated:

I couldn’t come to UCH [the usual tertiary hospital where the patient was receiving treatment for his chronic condition] throughout the lockdown]. I started coming again about two months ago (i.e., around March 2021). My primary health care was at a private hospital [P23, Female, 40s, Diabetes].

During the time I didn’t come to the hospital, I had a local doctor at a private clinic [P14, Male, in his 40s, hypertension].

#### Virtual consultation

Other patients adopted e-health approaches, such as virtual consultation. One of those who experienced this gave this account:

You won’t see any doctor. We have a family doctor. I used to complain [consult] on the phone, and he sent texts or consulted through WhatsApp audio or video calls. They will look at you and ask you to “touch where it’s paining you”. You cannot go to the hospital. All hospitals are locked. So, through WhatsApp, they now send a message to you that this is what you will buy, go to the pharmacy. Even at the pharmacy, they will give you something through the window. You can’t enter [P25, Male, 40s, diabetes mellitus].

#### Self-directed disease management

Some of the participants resorted to self-medicating, using medications that had been previously prescribed to them before the pandemic.

I was managing [my illnesses] based on the previous drugs that were given, some basic things that we have been told, “You don’t do this, you don’t do that.“ But regarding seeing them one-on-one, I was not seen” [P7, Male, 50s, hypertension].

## Discussion

In this study, we used the “Three Delays” model to describe the experience of people with chronic medical conditions accessing care during the pandemic. We also highlight how patients circumvent poor access to healthcare services. At the level of individual decision-making, delays were related to fear of contracting COVID-19 in the hospital, considered a hotspot of the COVID-19 pandemic. Regarding reaching an appropriate healthcare facility, delays mainly were attributed to the intra- and inter-city lockdowns, limiting the movements of persons. For those who successfully arrived at the healthcare facilities, delays were related to the unavailability of healthcare professionals, prioritization of COVID-19 patients, and mandatory adherence to COVID-19 protocols, including COVID-19 testing. To overcome the delays mentioned above, we found that people living with NCDs resorted to (i) using private healthcare facilities, which were more costly, (ii) using virtual consultation through mobile phone Apps and (iii) self-management, usually by repeating previously prescribed prescriptions to obtain medication.

In line with the “first delay” in the three-delay model, fear of contracting COVID-19 infection in the hospital constituted a significant barrier to healthcare service access for people with chronic diseases. The fear of contracting COVID-19 might have been associated with the widespread information about the high morbidity and mortality associated with COVID-19 infection among people with chronic conditions. Possibly, the possibility of contracting the infection outweighed the possible consequences of missing a doctor’s appointment. This fear was compounded by the fact that teaching hospitals in Nigeria were identified as major centres for COVID-19 care during the pandemic. Our findings are also supported by Malhotra et al. [33], who reported that many patients with chronic illnesses in Singapore voluntarily cancelled their medical appointments due to the fear of contracting COVID-19 infection. Nevertheless, the pandemic has pointed to flaws in traditional methods of disease follow-up and the need to develop better telehealth services for chronic care.

We found that during the COVID-19 era, with the lockdown restrictions in place, patients experienced delays in accessing care due to their inability to reach their usual healthcare facility. These travel restrictions constitute the second level of delay. Ideally, healthcare facilities should be close to people’s homes. However, with the limited availability of chronic care services in many Primary Health Care facilities in Nigeria [34], people with chronic illnesses must receive care at tertiary facilities. When patients live in highly congested cities, the tertiary facility becomes overwhelmed, and some patients must receive care in other cities with less congestion at the healthcare facilities. Similar findings were reported by Schwartz et al. [20] among Ugandan patients with hypertension who had difficulty accessing their medications at health facilities due to the COVID-19 lockdown. Hennelly et al. [35] also reported delays in healthcare access by Irish older adults during the COVID-19 pandemic. In such instances, alternative and other remote approaches to health care services should be implemented, such as virtual consultations and door-to-door delivery of medication packages.

The third delay in healthcare access was widespread during the pandemic and related to the inability of patients to access adequate healthcare after arriving at the care facility. Preliminary COVID-19 testing before receiving standard care was identified as a source of delay at healthcare facilities. Some patients with chronic conditions in the United States also experienced increased wait times at the emergency care unit because of the COVID-19 protocols [36]. We also found that appointments were rescheduled, and postponements were commonplace during COVID-19. Other studies involving people with chronic illnesses in the US, Europe, Israel, the UK, Italy, India, South Africa, and some developing countries have reported interruption in routine medical consultations with patients’ appointments postponed due to the pandemic [10, 12, 15, 16, 37–40]. Poor access to health care implies that many health systems will have to bear the burden of chronic illness complications.

Access to healthcare workers was also identified as a third-level delay by people with chronic diseases. Most healthcare personnel specializing in chronic disease care practice in Nigeria are in public teaching hospitals. Besides, health care is more affordable in government-owned than private hospitals. Hence, most patients with NCDs receive care at government-owned hospitals. During the COVID-19 pandemic, public hospitals became the center of COVID-19 treatment, causing a diversion of staff to COVID-19 services, thus reducing the number of patients accessing healthcare services [41]. In line with our findings, Rwandan patients also had to access care at private hospitals during the pandemic, as private hospitals were less affected [14]. Having to receive care at private facilities meant that most patients with NCDs had to pay more out-of-pocket and had reduced access to specialist care. Inadequate treatment can lead to complications, hence the need to diversify healthcare services and support private health facilities to provide adequate NCD services.

Self-directed disease management was another way people with chronic diseases circumvent the barriers to healthcare access during COVID-19. Self-directed disease management is understandable since they encountered difficulties accessing health care at the tertiary hospitals while the pharmacies were open. People with chronic diseases used their earlier prescriptions or empty drug containers to obtain medications. Although these must have helped in some ways, patients can underdose or overdose themselves since physicians sometimes adjust patients’ medication dosages during follow-up appointments. Our findings corroborate that of Lakshmi et al. [12], who reported that Indian patients with diabetes used the same previously prescribed medications for almost a year due to the COVID-19 travel restrictions. Self-medication was also practiced by some Kenyan healthcare workers with NCDs during the pandemic and was associated with adverse drug reactions [42]. During the COVID-19 pandemic, patients with NCDs, including diabetes and hypertension, in Pakistan avoided routine visits and resorted to self–medication [43]. Similarly, during the pandemic, patients with NCDs in Italy and Saudi Arabia were more prone to self-medication than the general population [38, 44]. Sensitization about the risks of self-medication and the development of alternative consultation methods, such as telemedicine or open-air consultation, could serve as short- and long-term solutions to the problem of self-medication.

Virtual consultation was found to be an alternative approach to seeking healthcare services, considering the challenges posed by the COVID-19 pandemic. Hence, it can be a beneficial alternative, as reported by some authors during the pandemic [45]. The use of smartphones for mobile health (mhealth) applications is increasing rapidly worldwide [46]. mhealth apps have been used to monitor health indicators (such as blood pressure, heart rate and glycosylated haemoglobin) and to support patients with diabetes [47]. Opportunities abound for using mhealth in Nigeria since many people use smartphones. Data in 2018 showed that between 25 and 40 million people in Nigeria use smartphones, predicted to increase to 140 million by 2025 [48]. The availability of smartphones remains relevant for developing and implementing mHealth programs to ensure a better outcome for patients with non-communicable diseases. In addition to virtual access to medical consultations, home delivery approaches such as those adopted during the COVID-19 pandemic for managing HIV could be adopted [49].

### Strengths and limitations

#### What this study adds

Using a qualitative method, this study provides unique data on the experience of people living with chronic non-communicable diseases in accessing health care during the COVID-19 pandemic. It also provides evidence of using an evolving alternative to physical consultation – mHealth - which should be further developed for NCD management. Additionally, we recommended strengthening primary and secondary healthcare facilities, including task-shifting to medical officers and using clinical protocols when shifting tasks.

#### Study limitations

The minimum six-month disease listed as an inclusion criterion might not be long enough to assess clinic appointments missed and behavior resulting from COVID pandemic movement restriction. Field notes were taken after the interview rather than during the interview. Retrospective notetaking may have caused recall bias, although all interviews were audio-recorded.

## Conclusion

People with chronic medical conditions had difficulty accessing healthcare services during the COVID-19 pandemic. They delayed their decision to use healthcare due to fear of COVID-19. Some experienced delays in transporting themselves due to the lockdown implementation, while others could not receive health care after arriving at the hospital, owing to the enforcement of COVID-19 screening protocols and the shortage of healthcare professionals to attend to them. The patients resorted to other means to circumvent the various delays they encountered. These strategies included using private healthcare facilities, self-medication, and virtual consultation. Alternative health service provision approaches, such as Mobile health apps that can help manage and prevent NCDs, should be considered in pandemic situations.

### Recommendations

Nigeria’s health system needs to be strengthened at the primary and secondary levels of care to reduce the pressure on tertiary healthcare facilities regarding managing NCDs. Patients with chronic conditions can receive follow-up care at primary healthcare centres. Hence, more efforts should be directed at providing material and human resources to ensure the capacity of PHCs to provide chronic healthcare services effectively. Catering for the possible absence of specialists, such as cardiologists and endocrinologists at the PHCs, requires task-shifting to medical officers being provided with evidence-based protocols to manage stable patients while other patients with complications are referred to tertiary facilities.

### Ethics considerations

The University of Ibadan/University College Hospital (UI/UCH) Institutional Review Committee gave the study ethical approval with approval number UI/EC/21/0065. Informed consent was obtained from all the participants and their legal guardians. The personal information of the participants was not collected to prevent breaking the ethical principles of anonymity. Besides, in preparing this manuscript, we have not included any information that might link the data collected from our participants to their identity. We ensured confidentiality by conducting the interviews privately - one-on-one with the interviewer. All aspects of the data collection were performed in accordance with the Declaration of Helsinki.

### Electronic supplementary material

Below is the link to the electronic supplementary material.

Supplementary Material 1

## Data Availability

Data are available upon reasonable request.
