# Supplementary material for: Access to healthcare services for people with non-communicable diseases during the COVID-19 pandemic in Ibadan, Nigeria: a qualitative study
Source: BMC Health Serv Res. 2023 Nov 9;23:1231. doi: 10.1186/s12913-023-10278-0 (PMC10636991; doi:10.1186/s12913-023-10278-0)
Supplement: Supplementary file 1 — Supplementary Material 1 [file 12913_2023_10278_MOESM1_ESM.docx]

Additional file 1

Covid-19 Vaccine: Access to healthcare services for people with Noncommunicable Diseases during the COVID-19 pandemic in Ibadan, Nigeria: A qualitative study.

**Introduction**

Good day and thank you for agreeing to participate in this interview.

As I mentioned when we first discussed over the phone, I would like to explore your thoughts about Covid-19 Vaccine. You have been invited to this discussion because you took part in an earlier study. We appreciate that you have given your time to be here with us.

There is no right or wrong answers. So, kindly tell me how you feel about the questions asked.

We will record this conversation to enable us write out the main point of the discussion after this episode. We will share a form with you to capture some information about you. Please fill appropriately and return to us before the end of the discussion. You do not need to mention your name or other information to identify you. We assure you of confidentiality and anonymity.

Thank you.

**Interview guide**

"Can you tell me about your experience and how life has been for you since COVID-19 started in Nigeria? What was access to healthcare like at UCH during the pandemic? (Probe: did you have your usual clinic appointment?) How did you manage your health during the COVID-19 lockdown? Would you say your health was affected because of the lockdown?

**Sociodemographic variables.**

1. Age (in years) as at last birthday ………..
2. Gender (a) Male (b) Female
3. Religion (a) Islam (b) Christianity (c) Traditional (d) Others
4. Marital status (a) Single (b) Engaged (c) Married (d) Divorced
5. Ethnicity (a) Yoruba (b) Hausa (c) Igbo (d) Others (specify)------------------
6. What is your highest level of education? a. Postgraduate degree (MSc or PhD)

b. Tertiary education: HND\BSc/B.Ed/B.A. c. Secondary School

d. Primary School e. No formal education
